# The causal relationship between immune cells and ankylosing spondylitis: a bidirectional Mendelian randomization study

**DOI:** 10.1186/s13075-024-03266-0

**Published:** 2024-01-16

**Authors:** Yuchang Fei, Huan Yu, Yulun Wu, Shanshan Gong

**Affiliations:** 1 Department of Integrated Chinese and Western Medicine, The First People’s Hospital of Jiashan, Jiashan Hospital Affiliated of Jiaxing University, Jiaxing, Zhejiang China; 2 The Department of Traditional Chinese Medicine, The First Affiliated Hospital of Ningbo University, Ningbo, Zhejiang China; 3 Center for Rehabilitation Medicine, Rehabilitation & Sports Medicine Research Institute of Zhejiang Province, Department of Rehabilitation Medicine, Zhejiang Provincial People’s Hospital, Affiliated People’s Hospital, Hangzhou Medical College, Hangzhou, Zhejiang China; 4 Department of Gastroenterology, The Third Affiliated Hospital of Zhejiang Chinese Medical University, Hangzhou, Zhejiang China

**Keywords:** Immune cell, Ankylosing spondylitis, Causal inference, Mendelian randomization

## Abstract

**Background:**

Ankylosing spondylitis (AS) is one of several disorders known as seronegative spinal arthritis (SpA), the origin of which is unknown. Existing epidemiological data show that inflammatory and immunological factors are important in the development of AS. Previous research on the connection between immunological inflammation and AS, however, has shown inconclusive results.

**Methods:**

To evaluate the causal association between immunological characteristics and AS, a bidirectional, two-sample Mendelian randomization (MR) approach was performed in this study. We investigated the causal connection between 731 immunological feature characteristic cells and AS risk using large, publically available genome-wide association studies.

**Results:**

After FDR correction, two immunophenotypes were found to be significantly associated with AS risk: CD14 − CD16 + monocyte (OR, 0.669; 95% CI, 0.544 ~ 0.823; *P* = 1.46 × 10^−4^; *P*_FDR_ = 0.043), CD33dim HLA DR + CD11b + (OR, 0.589; 95% CI = 0.446 ~ 0.780; *P* = 2.12 × 10^−4^; *P*_FDR_ = 0.043). AS had statistically significant effects on six immune traits: CD8 on HLA DR + CD8 + T cell (OR, 1.029; 95% CI, 1.015 ~ 1.043; *P* = 4.46 × 10^−5^; *P*_FDR_ = 0.014), IgD on IgD + CD24 + B cell (OR, 0.973; 95% CI, 0.960 ~ 0.987; *P* = 1.2 × 10^−4^;* P*_FDR_ = 0.021), IgD on IgD + CD38 − unswitched memory B cell (OR, 0.962; 95% CI, 0.945 ~ 0.980; *P* = 3.02 × 10^−5^; *P*_FDR_ = 0.014), CD8 + natural killer T %lymphocyte (OR, 0.973; 95% CI, 0.959 ~ 0.987; *P* = 1.92 × 10^−4^; *P*_FDR_ = 0.021), CD8 + natural killer T %T cell (OR, 0.973; 95% CI, 0.959 ~ 0.987; *P* = 1.65 × 10^−4^; *P*_FDR_ = 0.021).

**Conclusion:**

Our findings extend genetic research into the intimate link between immune cells and AS, which can help guide future clinical and basic research.

**Supplementary Information:**

The online version contains supplementary material available at 10.1186/s13075-024-03266-0.

## Introduction

Ankylosing spondylitis (AS) is a kind of spine arthritis that mostly affects the spine, sacroiliac joints, spinal attachment sites, and other axial bones, causing chronic inflammatory damage and loss of joint function [1]. AS is a chronic inflammatory autoimmune disease characterized clinically by severe pain, limited movement and spinal mobility abnormalities, and extra-skeletal organ consequences [2–4]. AS often affects young adults and is more common in men [5, 6], with HLA-B27 positive substantially related with more frequent attacks. The incidence and prevalence estimates vary from 0.05 to 1.4/10,000 people per year and 0.1 to 1.4%, respectively [7, 8]. NSAIDs, methotrexate, azathioprine, and biosynthetic disease-modifying anti-rheumatic medications (bDMARDs) are now effective therapy for systemic AS symptoms [6]. AS has a major influence on patients’ physical and mental health, resulting in enormous social expenses; nevertheless, early diagnosis and active intervention treatment can postpone and lessen the incidence of problems, as well as aid in improving the prognosis of AS.

AS is one of a group of disorders known as seronegative spinal arthritis (SpA), the origin of which is unknown. Existing epidemiological data show that inflammation plays an important role in the etiology of AS [8, 9], mostly through the interaction of hereditary and environmental variables that promote inflammation. MHC-encoded class I alleles, HLA-B27, endoplasmic reticulum aminopeptidase 1 (ERAP1), and IL-23R have all been linked to increased risk [10–12]. The immune system is made up of different immune cells, cytokines, and markers that control the immune response and inflammation. SpA is now described as a polygenic autoinflammatory illness in which innate immunological abnormalities can play a significant role, characterized by aberrant activation of innate and innate-like immune cells [13]. As high-throughput sequencing technology advances, there is a growing knowledge of the function of the immune system and critical cell types in the pathogenesis of AS. However, effective medicines have yet to be developed, and many patients experience problems or become unacceptable to existing medication therapies [14].

AS patients have immune system problems, and the percentage of CD8 + CD122 + T cells in peripheral blood is higher than in healthy controls, implying that CD8 + T cells may play a role in the pathogenesis and progression of AS [15]. Dendritic cells (DC) are important in AS. The illness can be transmitted through bone marrow transplantation, and DCs are thought to be the primary implicated cell [16]. Several investigations have found alterations in the function and gene expression of MD-DC from patients with human HLA B27 + central-axis SpA. AS patients have lower amounts of class II major tissue compatibility complex (MHC) molecules of human leukocyte antigen-DR (HLA-DR), which may result in decreased activity [17]. Several recent investigations have shed some light on B cell disease in AS patients. In individuals with AS, the number of CD27 + B cells is reduced, whereas the number of CD86 + and CD27 CD95 + B cells is raised [18]. The serum levels of IL-23 and IL-17 in the peripheral blood of AS patients are markedly elevated, which is linked to the disease [19, 20]. T cells, B cells, NK cells, and macrophages, as well as the IL-23/IL-17 axis, are all involved in the inflammatory development of AS [21–23]. Furthermore, results from other research have revealed that antibodies can be used as biomarkers to aid in the diagnosis and prognosis of ankylosing spondylitis. To date, however, the findings of studies on the causal association between AS and immunological inflammation have been inconsistent, which may be due to the small sample size used in the study design, confounding factors, and bias.

Mendelian randomization (MR) is an epidemiological etiological inference analysis approach based on the Mendelian independent distribution law [24, 25]. It uses genetic variation as an instrumental variable to infer if risk variables affect the outcomes. MR can lessen the possible bias induced by confounding and reverse causality when compared to other statistical methods [26, 27]. An association between immune cells and AS has been found in earlier observational studies [27, 28]. In this work, we conducted an extensive two-way two-sample MR analysis to elucidate the causal relationship between immune cell properties and AS.

## Materials and methods

### Study design

The causal link between 731 immune cells and AS was investigated using a two-sample MR analysis. The MR analysis seeks to clarify the causal link between single or multiple exposures and a single result by representing risk variables with genetic variation. It is worth mentioning that the instrumental variable (IV) must satisfy three fundamental assumptions: (1) genetic variation must be tightly tied to environmental exposure, (2) genetic variation is unrelated to potential environmental or genetic confounders of the result, and (3) the statistical strength of the link between genetic variation, exposure, and outcome may be estimated, and genetic variation only influences results via exposure pathways [29, 30]. This study’s exposure and result samples were human individuals, and it was a secondary analysis of already published data, and thus, no ethical approval was required. The Ethics Committee of the First People’s Hospital of Jiashan, Jiashan Hospital affiliated of Jiaxing University, granted us an exemption because all datasets utilized in this study were public domain.

### Genome-wide association study (GWAS) data sources for AS

From a comprehensive GWAS meta-analysis, we chose a genome-wide significant (*p* < 5 × 10^−8^) single nucleotide polymorphism (SNP) site related with AS as IV. The FinnGen database (https://www.finngen.fi/en/access_results) is used to collect and evaluate data from 500,000 Finnish biological sample library participants’ genomic and health data [31]. The study, which was based on the dataset finn-b-M13_ANKYLOSPON_STRICT, included 16,380,466 single nucleotide polymorphisms (SNPs), and GWAS was done on 218,030 Europeans (nCase = 599, nControl = 217,431).

### Immune cell GWAS data sources

A public catalog (GCST0001391 to GCST0002121) contains GWAS data for 731 immunophenotypes included in the study [32]. The data from 3757 Sardinians was used to calculate around 22 million genetic variations and validate relationships with autoimmune illnesses in the GWAS data on immunological characteristics. The GWAS aggregated data included 118 absolute cell counts (AC), 389 median fluorescence intensity (MFI) representing surface antigen levels, 32 morphological characteristics (MP), and 192 relative cell counts (RC). That is the ratio of cellular levels.

### Selection of instrumental variables (IVs)

According to recent studies, a loose cutoff value of *P* < 1 × 10^−5^ was used to select significant SNPs for various immune traits [33, 34]. Based on the European 1000 Genomes Project, the tool variables of *r*^2^ < 0.001 were excluded using the clump program in the PLINK software. If a sufficient number of IVs are missing from the resulting data, *r*^2^ > 0.8 is used as a proxy. In conducting reverse MR analysis, we chose a more stringent screening criterion, setting the significance threshold at 5 × 10^−8^ and *r*^2^ at 0.01. A total of 2475 independent IVs of immunophenotype were measured. Then, after removing IVs with low *F* statistics (< 10), 8 AS IVs were retained for further analysis.

### Statistical analysis

The R 4.2.1 software was used for all of the analyses. R packages “TwoSampleMR” and “MR-PRESSO” were used for MR analysis, and R was also used for data chart display.

We employed inverse variance weighted (IVW), MR-Egger, Weighted median (WM), and Weighted mode to assess the causal connection between 731 immunological characteristics and AS. To evaluate the bidirectional causal link between immunological characteristics and AS, the WM and MR multi-effect residual and outliers test (MR-PRESSO) were performed.

The major method of our MR research is IVW analysis, which is distinguished by ignoring the presence of an intercept term in regression and relying on outcome variance since it provides the most compelling estimation when the directed pleiotropy of IVs is missing [35]. To examine the heterogeneity of the selected IVs, Cochran’s *Q* statistic and accompanying *P*-values were utilized. If the null hypothesis is rejected, a random effect IVW rather than a fixed effect IVW is utilized [36]. MR-Egger is a Mendelian randomization approach that summarizes data to test the robustness of outcomes [37]. As sensitivity analyses for Mendelian randomization studies with many genetic variants, both MR-Egger and WM should be regarded as supplements. The MR-PRESSO approach is used to find horizontal pleiotropy outliers in multi-instrument summary-level MR tests and re-evaluate causal effects after pleiotropy removal IV [38]. Furthermore, the scatter plot reveals that outliers have little effect on the results. The funnel plot depicts the correlation’s robustness in the absence of heterogeneity.

## Results

### Exploring the impact of immune phenotype on the causal relationship of AS

**Fig. 1:**
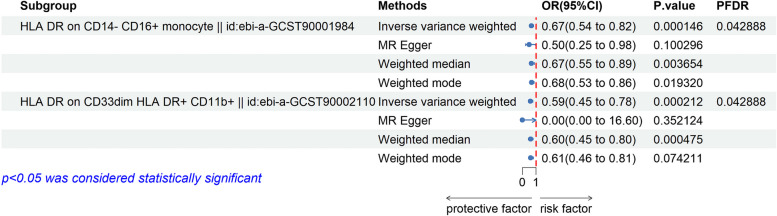
The forest map shows a causal relationship between immune traits and AS. IVW, inverse variance weighting; CI, confidence interval

As the primary analysis methods, we employed the two-sample MR analysis and IVW approach to look into the causal association between immunophenotype and AS. Cochran’s *Q* test was used to evaluate data heterogeneity, and the fixed effect model IVW was used when *P* > 0.05. After FDR test adjustment (*P*_FDR_ < 0.05), two immunophenotypes were found to have a causal relationship to AS, which were protective factors: CD14 − CD16 + monocyte (monocyte group) and CD33dim HLA DR + CD11b + (bone marrow cell group). Among them, the odds ratio (OR) of CD14 − CD16 + monocyte against AS calculated by IVW method was 0.669 (95% CI, 0.544 ~ 0.823; *P* = 1.46 × 10^−4^; *P*_FDR_ = 0.043, Table 1). Results from other MR methods were similar: weighted mode (OR, 0.676; 95% CI, 0.531–0.861; *P* = 0.019), weighted median (OR, 0.670; 95% CI, 0.550 ~ 0.890; *P* = 3.6 × 10^−3^), and MR-Egger (OR, 0.499; 95% CI, 0.254–0.982; *P* = 0.100). The risk ratio of CD33dim HLA DR + CD11b + to AS was estimated to be 0.589 (95% CI, 0.446 ~ 0.780; *P* = 2.12 × 10^−4^; *P*_FDR_ = 0.043, Table 1). The results of the other three methods were similar: weighted median (OR, 0.602; 95% CI, 0.452 ~ 0.800; *P* = 4.75 × 10^−4^), weighted mode (OR, 0.607; 95% CI, 0.457 ~ 0.805; *P* = 0.074), and MR-Egger (OR, 1.56 × 10^−6^; 95% CI, 1.46 × 10^−13^ ~ 16.601; *P* = 0.352). Although *P* > 0.05 of MR-Egger, the beta values of the above four analysis methods are all > 0, that is, they are all in the same direction. The largest distinction between the MR-Egger method and IVW, according to earlier statistical research reports [39, 40], is that the MR-Egger method takes into account the existence of an intercept term in regression. This term is used to measure the average polymorphism size among instrumental variables, whereas the slope provides an unbiased estimate of the causal effect. Generally speaking, the IVW method’s standard error is less than the MR-Egger method’s. Therefore, the results of IVW, the gold standard of MR analysis, will be employed preferentially in the absence of heterogeneity and horizontal pleiotropy. In summary, the MR analysis is still statistically significant. There is no statistical difference between the egger_intercept of MR-Egger and 0 (*P*val > 0.05, Supplementary Table 3), so we can assume that there is no horizontal pleiotropy. Sensitivity analysis further demonstrated the robustness of the causal associations derived from the analysis (Table 2, Fig. 1).

**Table 1:** Causal effects of immune cells on ankylosing spondylitis

Exposure	Outcome	Method	nSNP	*P*	OR (95% CI)
HLA DR on CD14 − CD16 + monocyte	Ankylosing spondylitis	Inverse variance weighted	7	1.46E − 04	0.669 (0.544,0.823)
		MR-Egger	7	0.100	0.499 (0.254,0.982)
		Weighted median	7	0.004	0.670 (0.550,0.890)
		Weighted mode	7	0.0193	0.676 (0.531,0.861)
HLA DR on CD33dim HLA DR + CD11b +		Inverse variance weighted	3	2.12E − 04	0.589 (0.446,0.780)
		MR-Egger	3	0.352	0.00000156 (1.45E − 13,16.601)
		Weighted median	3	4.75E − 04	0.602 (0.452,0.800)
		Weighted mode	3	0.074	0.607 (0.457,0.805)

**Table 2:** Sensitivity analysis results of causal effects of immune cells on ankylosing spondylitis

Exposure	Outcome	Method	*Q*	Q_df	Q_*P*val
HLA DR on CD14 − CD16 + monocyte	Ankylosing spondylitis	MR-Egger	6.242	5	0.283
		Inverse variance weighted	7.237	6	0.299
HLA DR on CD33dim HLA DR + CD11b +		MR-Egger	0.195	1	0.659
		Inverse variance weighted	2.615	2	0.27

### Exploring the impact of AS on the causal relationship of immune phenotype

**Fig. 2:**
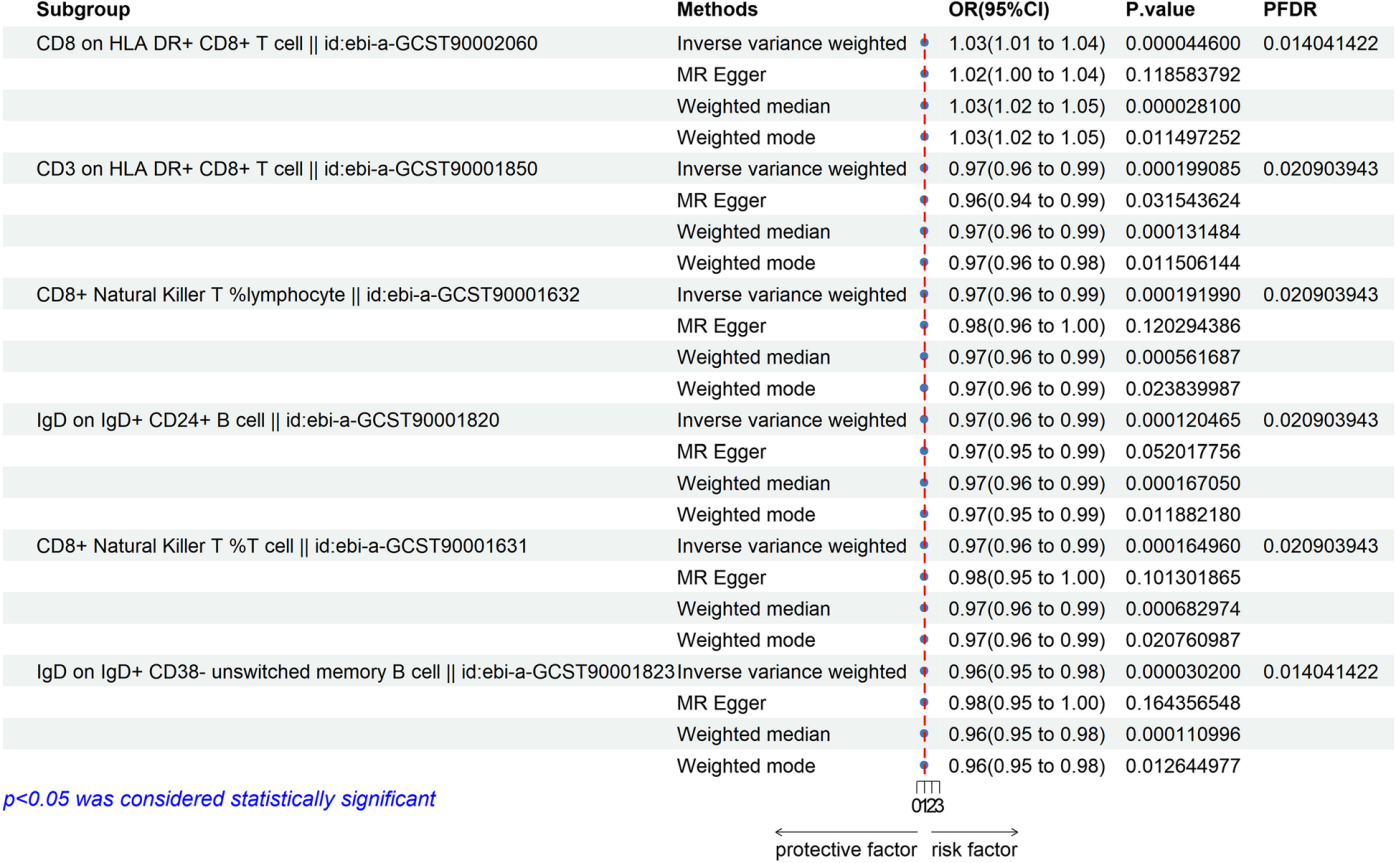
Forest maps with different methods show a causal relationship between AS and immune traits. IVW, inverse variance weighting; CI, confidence interval

After FDR adjustment (*P*_FDR_ < 0.05), a total of six immune traits were detected, and the difference was statistically significant (*P* < 0.05). Among them, AS was a risk factor for 1 immunophenotype: CD8 on HLA DR + CD8 + T cell (T cell group), and AS was a protective factor for 5 immunophenotypes, respectively: one T cell (CD3 on HLA DR + CD8 + T cell), two B cells (IgD on IgD + CD24 + B cell, IgD on IgD + CD38 − unswitched memory B cell), and two types of NKT cells (CD8 + natural killer T %lymphocyte, CD8 + natural killer T %T cell). Data heterogeneity was assessed by Cochran’s *Q* test (*P* > 0.05), and fixed effect model IVW was used in all cases. Among them, the OR of AS against CD8 on HLA DR + CD8 + T cell calculated by IVW method was 1.029 (95% CI, 1.015 ~ 1.043; *P* = 4.46 × 10^−5^; *P*_FDR_ = 0.014, Tables 3 and 4). Results from other MR methods were similar: weighted mode (OR, 1.031; 95% CI, 1.015–1.047; *P* = 0.011), weighted median (OR, 1.031; 95% CI, 1.016 ~ 1.045; *P* = 2.81 × 10^−5^), and MR-Egger (OR, 1.022; 95% CI, 1.000 ~ 1.045; *P* = 0.119). The risk ratio of AS to IgD on IgD + CD24 + B cell was estimated to be 0.973 (95% CI, 0.960 ~ 0.987; *P* = 1.2 × 10^−4^; *P*_FDR_ = 0.021, Table 3). The results of the other three methods were similar: weighted median (OR, 0.972; 95% CI, 0.958 ~ 0.987; *P* = 1.67 × 10^−4^), weighted mode (OR, 0.970; 95% CI, 0.955 ~ 0.985; *P* = 0.012), and MR-Egger (OR, 0.970; 95% CI, 0.950–0.991; *P* = 0.052). The risk ratio of AS to IgD on IgD + CD38 − unswitched memory B cell was estimated to be 0.962 (95% CI = 0.945 ~ 0.980; *P* = 3.02 × 10^−5^, *P*_FDR_ = 0.014, Table 3). The results of the other three methods were similar: weighted median (OR, 0.964; 95% CI, 0.946 ~ 0.982; *P* = 1.11 × 10^−4^), weighted mode (OR, 0.964; 95% CI, 0.946–0.982; *P* = 0.013), and MR-Egger (OR, 0.976; 95% CI, 0.948 ~ 1.003; *P* = 0.164). The risk ratio of AS for CD8 + natural killer T %lymphocyte was estimated to be 0.973 (95% CI, 0.959 ~ 0.987; *P* = 1.92 × 10^−4^; *P*_FDR_ = 0.021, Supplementary Table S2). The other three methods had similar results: weighted median (OR, 0.974; 95% CI, 0.959 ~ 0.989; *P* = 5.62 × 10^−4^), weighted mode (OR, 0.974; 95% CI, 0.959 ~ 0.990; *P* = 0.024), and MR-Egger (OR, 0.978; 95% CI, 0.956 ~ 0.999; *P* = 0.120). The risk ratio of AS to CD8 + natural killer T %T cell was estimated to be 0.973 (95% CI, 0.959 ~ 0.987; *P* = 1.65 × 10^−4^; *P*_FDR_ = 0.021, Table 3). The results of the other three methods were similar: weighted median (OR, 0.973; 95% CI, 0.958 ~ 0.989; *P* = 6.83 × 10^−4^), weighted mode (OR, 0.974; 95% CI, 0.959 ~ 0.989; *P* = 0.021), and MR-Egger (OR, 0.976; 95% CI, 0.955 ~ 0.998; *P* = 0.101). Although *P* > 0.05 of MR-Egger, the beta values of the above four analysis methods are all in the same direction, so the MR analysis is still statistically significant. There is no statistical difference between the egger_intercept of MR-Egger and 0 (*P*val > 0.05, Supplementary Table S4), so we can assume that there is no horizontal pleiotropy. Sensitivity analysis further demonstrated the robustness of the causal associations derived from the analysis (Supplementary Table S4, Fig. 2).

**Table 3:** Causal effects of ankylosing spondylitis on immune cells

Exposure	Outcome	Method	nSNP	*P*	OR (95% CI)
Ankylosing spondylitis	CD8 on HLA DR + CD8 + T cell	Inverse variance weighted	6	4.46E − 05	1.029 (1.015, 1.043)
		MR-Egger	6	0.119	1.022 (1.000, 1.045)
		Weighted median	6	2.81E − 05	1.031 (1.016, 1.045)
		Weighted mode	6	0.011	1.031 (1.015, 1.047)
	CD3 on HLA DR + CD8 + T cell	Inverse variance weighted	6	1.99E − 04	0.974 (0.961, 0.988)
		MR-Egger	6	0.032	0.964 (0.943, 0.986)
		Weighted median	6	1.31E − 04	0.971 (0.956, 0.986)
		Weighted mode	6	0.012	0.970 (0.955, 0.985)
	CD8 + natural killer T %lymphocyte	Inverse variance weighted	6	1.92E − 04	0.973 (0.959, 0.987)
		MR-Egger	6	0.120	0.978 (0.956, 0.999)
		Weighted median	6	5.62E − 04	0.974 (0.959, 0.989)
		Weighted mode	6	0.024	0.974 (0.959, 0.990)
	IgD on IgD + CD24 + B cell	Inverse variance weighted	6	1.20E − 04	0.973 (0.960, 0.987)
		MR-Egger	6	0.052	0.970 (0.950, 0.991)
		Weighted median	6	1.67E − 04	0.972 (0.958, 0.987)
		Weighted mode	6	0.012	0.970 (0.955, 0.985)
	CD8 + natural killer T %T cell	Inverse variance weighted	6	1.65E − 04	0.973 (0.959, 0.987)
		MR-Egger	6	0.101	0.976 (0.955, 0.998)
		Weighted median	6	6.83E − 04	0.973 (0.958, 0.989)
		Weighted mode	6	0.021	0.974 (0.959, 0.989)
	IgD on IgD + CD38 − unswitched memory B cell	Inverse variance weighted	6	3.02E − 05	0.962 (0.945, 0.980)
		MR-Egger	6	0.164	0.976 (0.948, 1.003)
		Weighted median	6	1.11E − 04	0.964 (0.946, 0.982)
		Weighted mode	6	0.013	0.964 (0.946, 0.982)

**Table 4:** Sensitivity analysis results of causal effects of ankylosing spondylitis on immune cells

Exposure	Outcome	Method	*Q*	Q_df	Q_*P*val
Ankylosing spondylitis	CD8 on HLA DR + CD8 + T cell	MR-Egger	1.329	4	0.856
		Inverse variance weighted	1.927	5	0.859
	CD3 on HLA DR + CD8 + T cell	MR-Egger	2.495	4	0.645
		Inverse variance weighted	3.82	5	0.576
	CD8 + natural killer T %lymphocyte	MR-Egger	0.91	4	0.923
		Inverse variance weighted	1.259	5	0.939
	IgD on IgD + CD24 + B cell	MR-Egger	3.197	4	0.525
		Inverse variance weighted	3.337	5	0.648
	CD8 + natural killer T %T cell	MR-Egger	0.732	4	0.947
		Inverse variance weighted	0.912	5	0.969
	IgD on IgD + CD38 − unswitched memory B cell	MR-Egger	2.853	4	0.583
		Inverse variance weighted	4.321	5	0.504

## Discussion

This study examined the relationship between 731 immune cells and AS using a large number of publicly available genetic data. This is the first 2-way MR analysis to investigate the link between several immunophenotypes and novel AS. We discovered a strong causal connection between 2 immunophenotypes for AS (*P*_FDR_ < 0.05) and AS for 6 immunophenotypes (*P*_FDR_ < 0.05) in this study.

According to this study, the risk of AS decreased as the proportion of CD14 − CD16 + monocytes increased. Monocytes are blood cells that help maintain vascular homeostasis and act as early warning signs of pathogens in acute infections, whereas CD14 − CD16 + monocytes are unusual [41]. Non-classical monocytes, which serve as the initial line of defense against pathogens, are widely thought to have anti-inflammatory properties and have been demonstrated to be protective and favorably connected with disease load [42, 43]. A significant negative connection has been found between the concentration of non-classical monocytes and the number of swollen joints in SpA patients [44]. Existing research indicates that the cytokines released by it play a function in the disease process of AS [45]. Patients with AS had considerably higher plasma IL-1β concentrations in the Chinese community [46]. TNF − levels are higher in AS patients than in normal patients, and TNF − levels can be employed as one of the major indicators for the supplementary diagnosis of AS and the evaluation of disease activity [47]. IFN − may cause an unfolded protein response (UPR) in AS cells expressing HLA-B27 [48]. CD33dim, HLA-DR, and CD11b + are progranulocytes, late myelocytes, and proto-single cells generated from normal myeloid cell lines, respectively. HLA-DR may be related with more severe peripheral joint involvement in AS patients, according to research [49]. In Mexicans, HLA-DR is strongly related with SpA but not with B27, and HLA-DR1 is associated with an older age of onset [50]. Other research, however, have revealed that HLA-DR has a minimal influence on AS and that the influence on the severity of AS disease is not statistically significant [51].

Furthermore, this study discovered that AS is related with lower CD8 levels on CD8 + HLA-DR + T cells, as well as CD3 on HLA DR + CD8 + T cells, IgD + CD24 + B cell, IgD + CD38 − unswitched memory B cell, and CD8 + natural killer T %lymphocyte. Natural CD8 + HLA-DR + T cells have immunosuppressive qualities, according to research [52], and CD8 + HLA-DR + regulatory T cells can block immune response via the PD-1/PD-L1 axis [53]. This may provide clues to similar pathways of involvement in autoimmune control. However, the existing literature has not found changes in the expression level of CD8 + HLA-DR + T cells in AS, which may be worth further exploration. CD24( +) B cells in AS patients are defective in inhibiting the activation of naive and memory CD8( +) T cells [54] Clinical studies have found that the percentage of natural killer T (NKT) cells in the peripheral blood of AS patients is higher than that of healthy controls [55]. Regretfully, NKT cell expression subgroup analysis in AS patients is not currently available. Furthermore, a recent meta-analysis produced the opposite conclusion, finding no statistically significant difference in the percentage of NKT cells between patients with AS and healthy controls [56].

Our two-sample MR study was a statistical analysis with a huge sample size and strong statistical efficacy that used the large GWAS cohort already in existence, which included more than 220,000 AS patients and European populations with immunological features. To determine the causal association between these genetic variants and outcomes based on the genetic variability associated with environmental factors, MR is utilized as an epidemiological study tool. Since exogenous confounding variables have no effect on genetic variation, the measurement error of genetic variation and its effect is negligible, and the findings are generally trustworthy. Our study is not without limits, though. First off, the finding is still somewhat limited in its applicability because the GWAS data used in this study was limited to the European population. Second, in order to acquire more precise analysis results, we are unable to perform additional population subgroup stratification analysis of the current AS population because the GWAS data currently do not contain individual patient information. Third, this study uses a two-way MR analysis. While it will be very helpful in resolving the causal network direction issue [57], biological mechanisms must also be taken into consideration when interpreting MR results; statistical effect values alone cannot provide this information. The substantial causal relationship between immune features and AS will be more thoroughly investigated if more robust biological evidence can be added to support it, as there are currently insufficient in-depth investigations of some immune traits in AS patients with favorable results.

## Conclusion

In conclusion, we further elucidated the pattern of interaction between AS and the immune system and investigated the causal association between several immunophenotypes and AS using two-way MR analysis. Two-way MR analysis was employed in our work to lessen reverse causality. The accuracy of causal inference is further enhanced and the impact of additional confounding factors is diminished. In addition to offering useful value hints for the treatment of AS, this will provide compelling genetic data to support investigations into the pathophysiology and biological function of AS.

### Supplementary Information

**Additional file 1:** **Figure S1.** Scatter plot of causality of immune cells on AS. (A) HLA DR on CD14- CD16+ monocyte, (B) HLA DR on CD33dim HLA DR+ CD11b+. AS, Ankylosing Spondylitis.

**Additional file 2:** **Figure S1.** Scatter plot of causality of AS on immune cells. (A) CD8 on HLA DR+ CD8+ T cell, (B) CD3 on HLA DR+ CD8+ T cell, (C) CD8+ Natural Killer T %lymphocyte, (D) IgD on IgD+ CD24+ B cell, (E) CD8+ Natural Killer T %T cell, (F) IgD on IgD+ CD38- unswitched memory B cell. AS, Ankylosing Spondylitis.

**Additional file 3:** **Supplementary Table S1.** Summary of MR Analysis results of causal relationship between immune cells and ankylosing spondylitis.

**Additional file 4:** **Supplementary Table S2.** Summary of MR Analysis results of causal relationship between ankylosing spondylitis and immune cells.

**Additional file 5:** **Supplementary Table S3.** Pleiotropy analysis results of causal effects of immune cells on Ankylosing spondylitis.

**Additional file 6:** **Supplementary Table S4.** Pleiotropy analysis results of causal effects of Ankylosing spondylitis on immune cells.

## Data Availability

No datasets were generated or analysed during the current study.

## Abbreviations

AS: Ankylosing spondylitis
SpA: Seronegative spinal arthritis
MR: Mendelian randomization
bDMARDs: Biosynthetic disease-modifying anti-rheumatic medications
ERAP1: Endoplasmic reticulum aminopeptidase 1
DC: Dendritic cells
MHC-II: Class II major tissue compatibility complex
HLA-DR: Human leukocyte antigen-DR
IV: Instrumental variable
GWAS: Genome-wide association study
SNP: Single nucleotide polymorphism
AC: Absolute cell counts
MFI: Median fluorescence intensity
MP: Morphological characteristics
RC: Relative cell counts
IVW: Inverse variance weighted
WM: Weighted median
CI: Confidence interval
MR-PRESSO: Multi-effect residual and outliers test
OR: Odds ratio
UPR: Unfolded protein response
NKT: Natural killer T
