# Supplementary figures and images for: The causal relationship between immune cells and ankylosing spondylitis: a bidirectional Mendelian randomization study
Source: Arthritis Res Ther. 2024 Jan 16;26:24. doi: 10.1186/s13075-024-03266-0 (PMC10790477; doi:10.1186/s13075-024-03266-0)

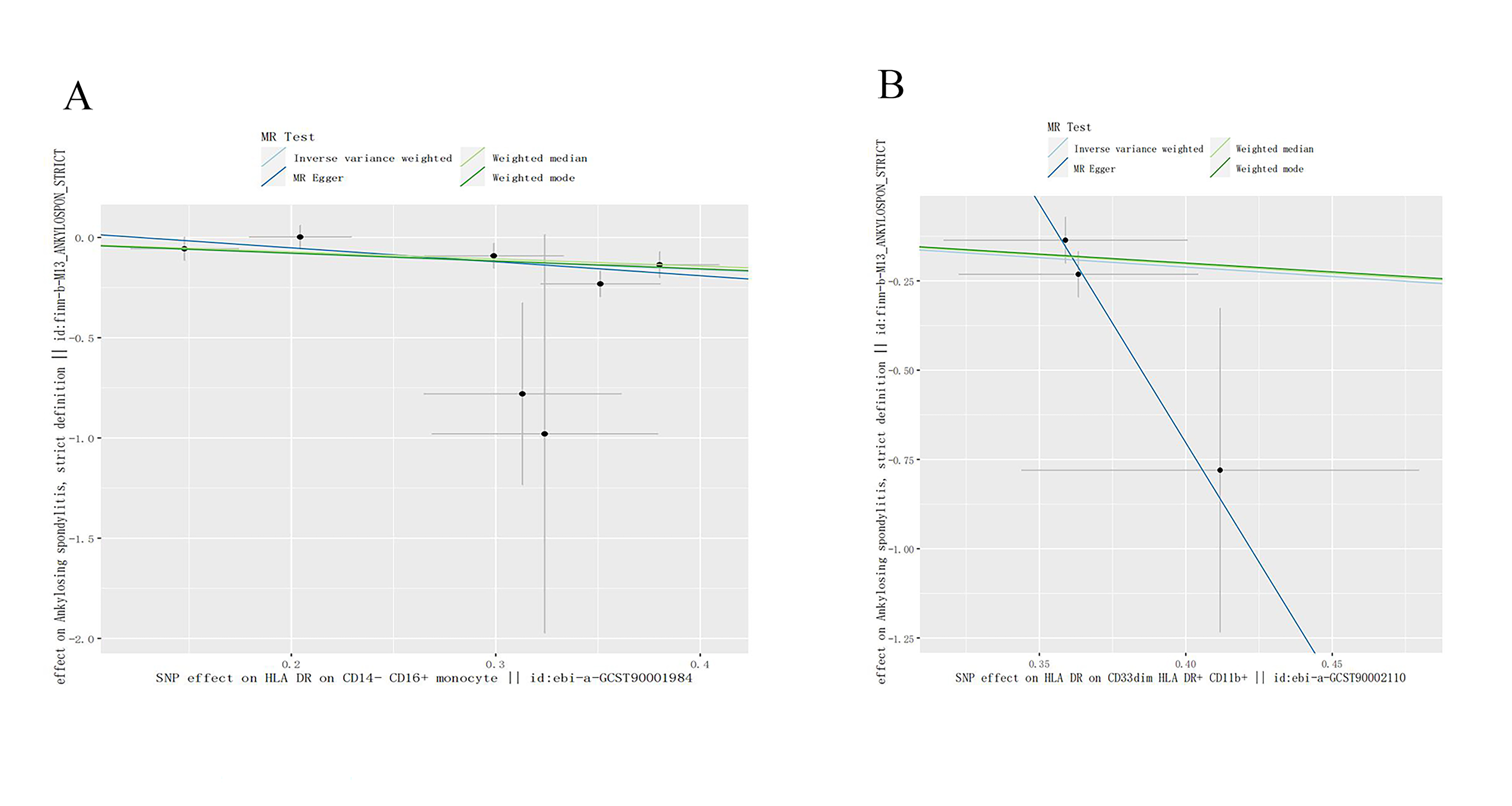

Supplement: Supplementary file 1 — Additional file 1: Figure S1. Scatter plot of causality of immune cells on AS. (A) HLA DR on CD14- CD16+ monocyte, (B) HLA DR on CD33dim HLA DR+ CD11b+. AS, Ankylosing Spondylitis. [file 13075_2024_3266_MOESM1_ESM.jpg]
